# Priority setting: what constitutes success? A conceptual framework for successful priority setting

**DOI:** 10.1186/1472-6963-9-43

**Published:** 2009-03-05

**Authors:** Shannon L Sibbald, Peter A Singer, Ross Upshur, Douglas K Martin

**Affiliations:** 1 Department of Health Policy, Management and Evaluation, University of Toronto, Toronto, Canada; 2 University of Toronto Joint Centre for Bioethics, Toronto, Canada; 3 The McLaughlin-Rotman Centre for Global Health, Toronto, Canada; 4 Sunnybrook Health Science Centre, Toronto, Canada

## Abstract

**Background:**

The sustainability of healthcare systems worldwide is threatened by a growing demand for services and expensive innovative technologies. Decision makers struggle in this environment to set priorities appropriately, particularly because they lack consensus about which values should guide their decisions. One way to approach this problem is to determine what all relevant stakeholders understand successful priority setting to mean. The goal of this research was to develop a conceptual framework for successful priority setting.

**Methods:**

Three separate empirical studies were completed using qualitative data collection methods (one-on-one interviews with healthcare decision makers from across Canada; focus groups with representation of patients, caregivers and policy makers; and Delphi study including scholars and decision makers from five countries).

**Results:**

This paper synthesizes the findings from three studies into a framework of ten separate but interconnected elements germane to successful priority setting: stakeholder understanding, shifted priorities/reallocation of resources, decision making quality, stakeholder acceptance and satisfaction, positive externalities, stakeholder engagement, use of explicit process, information management, consideration of values and context, and revision or appeals mechanism.

**Conclusion:**

The ten elements specify both quantitative and qualitative dimensions of priority setting and relate to both process and outcome components. To our knowledge, this is the first framework that describes successful priority setting. The ten elements identified in this research provide guidance for decision makers and a common language to discuss priority setting success and work toward improving priority setting efforts.

## Background

Priority setting, also known as rationing or resource allocation, is a complex and difficult problem faced by all decision makers at all levels of all health systems, including macro (e.g. governments), meso (e.g. regional health authorities (RHAs), hospitals), and micro (e.g. clinical programs) levels. There is relatively little interaction between decision makers at the three levels, or among institutions, regarding the setting of priorities. Consequently, priority setting has been described as a series of unconnected experiments with no systematic mechanism for capturing the lessons, or evaluating the strengths and weaknesses, of each experiment [1]. Hospital administrators, constrained by budget restrictions and confronted by increasing demand, find it a particularly difficult challenge to maintain services and quality, while controlling costs; decision makers (or leaders) *lack *guidance and information for priority setting and are unaware of priority setting tools available to them [2-4]. Mitton and Donaldson found decision makers were "frustrated with the lack of an explicit priority setting framework" and questioned "the credibility of resource allocation decision-making" ([4]p. 1660). Several studies have reported that leaders desire an explicit framework to guide priority setting [4-6] and acknowledged leadership as a key area where improvement can make the most difference [7].

The sustainability of healthcare systems worldwide is threatened by a growing demand for services and expensive innovative technologies. Decision makers struggle in this environment to set priorities appropriately, particularly because they lack consensus about which values should guide their decisions [8]. One way to approach this problem is to determine what relevant stakeholders understand successful priority setting to mean. Greater insight into stakeholders' attitudes and perceptions of achieving successful priority setting could improve the way in which institutions and healthcare organizations set priorities.

Successful priority setting is a desirable goal for decision makers; however there is no agreed upon definition for successful priority setting, so there is no way of knowing if an organization achieves it. Priority setting is extremely complex – choosing between competing values makes priority setting fundamentally an ethical issue [9]. Different disciplines offer their own perspective on how priority setting 'ought' to be done, defining 'good' (or successful) priority setting through values such as efficiency, equity, or justice. Discipline specific approaches and priority setting frameworks can help decision makers with priority setting: health economics encourages a focus on efficiency, policy approaches focus on legitimacy, evidence-based medicine looks to effectiveness. Daniels and Sabin created 'accountability for reasonableness' (A4R) with legitimacy and fairness as two key goals of priority setting [10]. Interdisciplinary approaches are also available such as program-budgeting and marginal analysis (PBMA)[11], health technology assessment (HTA)[12]. Menon *et *al. described priority setting in four steps: (1) identification of health care needs, (2) allocation of resources, (3) communication of decisions to stakeholders, and (4) management of feedback from them [13]. Still, there is no consensus that any one framework provides the 'correct' or 'best' comprehensive definition of successful priority setting.

These normative approaches are necessary because they help identify important values and underlying assumptions in priority setting, however alone they are insufficient and provide only a piece of a definition of successful priority setting. The problem that remains is: priority setting involves the adjudication between many relevant values and that people (and disciplines) will disagree about which values should dominate in any specific priority setting context and there is no agreed upon normative approach for resolving the disagreement. When relevant values conflict, decision makers must rely on developing context-specific agreement in order to achieve priority setting success.

Empirical studies are also important for understanding current decision making practices within healthcare organizations [14,15]; since they identify current priority setting practices, they provide insight into defining successful priority setting. In recent years, there have been empirical descriptions of priority setting in various contexts (e.g. waitlists [16-18], hospitals [19-21], and RHAs [13,22]). Other empirical studies have evaluated actual priority setting against an ethical framework (e.g. [19,23]). Studies have been done detailing factors that influence priority setting practices, including technical factors (such as clinical practice guidelines), non-technical factors (such as alignment with goals) [13] and clusters of factors [24]. Several studies have brought forth components for improving priority setting or ensuring success in priority setting, such as stakeholder engagement [13] increased dialogue [4], a culture supporting explicit priority setting [6], decision maker/group composition (size and representation) [25], clear information management and clarity of process [5], and local ownership and awareness of local politics [26].

Only a few studies have presented ideas for evaluating the success of priority setting including: economic evaluations [27,28], checklists looking at both pragmatic (such as establish organizational objectives and ensure implementation) and ethical considerations (such as publicity and appeals)[29], success parameters (effect on organizational priorities and budgets, effect of staff, and effect on community, efficiency of priority setting process, fairness, conformity with conditions of accountability for reasonableness)[2], a criteria-based framework (including objectives and context, methodology, process issues, and study outcomes)[30], outputs-based measures (such as usefulness re-allocation, improved patient outcomes) [31], and a model for ethical standards (including health of patients, professional (clinical) expertise, public health, unmet health needs, advocacy for social policy reform, relationships of special ethical importance (with employees), organizational solvency/survival, and benefit to community) [32]. Together, these studies contribute to our understanding of successful priority setting; but on their own, do not provide a comprehensive definition.

Evaluating success of health care (and other sectors) is possible through many of the aforementioned tools/processes, and different instruments may elicit different results [33]. The problem with these studies is their limited focus (narrow organizational study and/or small range of stakeholders). While we are more cognizant of important factors in successful priority setting, we still do not have a complete picture of it.

Normative approaches tell us what ought to be done, empirical studies tell us what is being done, and we are still left with a lack of consensus on an appropriate approach to successful priority setting. There is a need to define successful priority setting, to provide a common language, and to come to some agreement on conceptual basis for the concept.

A first step to ground such a definition is to collect and synthesize the views of stakeholders with direct priority setting knowledge and experience. Stakeholders include decision makers (particularly in publicly funded health systems, who are under growing pressure to base their decisions on available evidence and to demonstrate the effectiveness of their decision), patients (since the health system exists for them and because they fund the health system through taxes, insurance premiums or out-of-pocket payments), and priority setting scholars (who can provide different theoretical viewpoints on decision making). Creating a framework that defines success in priority setting is a necessary step toward improving priority setting practices in healthcare organizations [34,35].

The purpose of this paper is to present a synthesized definition of successful priority setting brought together from the findings of three empirical studies describing successful priority setting from the viewpoint of stakeholders (decision makers, patients, and priority setting scholars). The definition is presented as a conceptual framework with ten elements. The framework we describe here is a new development for evaluating priority setting; it can provide guidance to decision makers and scholars interested in successful priority setting.

## Methods

The findings reported here were derived from three separate but related empirical studies that used different data collection methods, but similar data analysis techniques. The overarching goal for the three studies was to create a conceptual framework for achieving successful priority setting. Study 1 gathered international perspectives through a Delphi consensus building initiative [36]. Study 2 used qualitative interviews to capture the views of decision makers across the Canadian healthcare system. Study 3 included the perspective of Canadian public/patients and policy makers and used multiple interconnected focus groups called the "circle within a circle" approach (table 1) [37]. Each study used a unique set of participants; there was no overlap. By bringing these three data sets together, we tapped into a diverse and rich knowledge base and captured what we feel to be a comprehensive description of successful priority setting. What follows is a combined description of the methods for all three studies.

**Table 1:** Description of Study 3 Focus Group Design

Study 3 used a distinctive focus group design called a circle-within-a-circle. A total of five focus groups were held. First, two independent focus groups were held, one with patients and one with policy makers. Second, two additional focus groups were held using the "circle within a circle" approach: the first had the patients on the inside and the decision makers on the outside, the second had the opposite (decision makers on the inside). The final focus group had both groups participating, sitting side-by-side in a large circle. This approach permitted data collection with stakeholder group that may have had problems due to power imbalance; for example, group-specific issues could be explored in depth, as well as providing an invaluable opportunity for knowledge exchange.	

### Participants

Study 1 (Delphi panel) consisted of 12 priority setting scholars and healthcare decision makers from five different health systems (table 2), chosen for their experience and interest in priority setting (i.e., published work in the field, different disciplinary approaches to priority setting and international perspectives).

**Table 2:** Summary of Delphi Panelists

	**Participant Role**	**Country**
1	Decision Maker	Canada

2	Decision Maker	Norway

3	Decision Maker	Norway

4	Decision Maker	U.K.

5	Decision Maker	U.S.A.

6	Scholar	Canada

7	Scholar	Canada

8	Scholar	Canada

9	Scholar	Norway

10	Scholar	U.K.

11	Scholar	U.S.A.

12	Scholar	Uganda

Study 2 consisted of senior or executive level decision makers in healthcare organizations across Canada sampled using two methods: (1) theoretical sampling – people who were involved in a significant aspect of priority setting and (2) 'snowball' sampling – participants were asked to identify others (colleagues) who might have knowledge or insight into priority setting and who should be interviewed. Participants were sought out until conceptual saturation was reached (i.e. until no new concepts were identified in successive interviews). Participants came from 45 different organizations with representation from every province except Newfoundland and Prince Edward Island (table 3). Attempts were also made to ensure there was representation within provinces – interviews did not focus solely on the capital regions of each province.

**Table 3:** Summary of Interview Participants

***MACRO***	Provincial Ministry of Health *(British Columbia – 1; Alberta – 1; Saskatchewan – 1; Ontario – 1, New Brunswick – 1; Nova Scotia – 2)*	7
***MESO***	Hospital Senior Management (*British Columbia – 2; Alberta – 1; Ontario – 12, Quebec – 2; Nova Scotia – 1*)	18
	
	Senior Management of Community Care Access Centres in Ontario	3
	
	Senior Management and Board Members of Regional Health Authorities (*British Columbia – 1; Alberta – 6; Saskatchewan – 3*)	10
	
	Senior Management of Private Health Care Organizations (*Alberta*)	2
	
	Directors/Executive Directors of District Health Councils (3) and Public Health Units (2) (*Ontario*)	5

***MICRO***	Clinician Managers in hospitals (*Alberta – 4, Saskatchewan – 1; Ontario – 1; Quebec – 1; Nova Scotia – 1*)	8
	
	Other (policy analyst/consultants, ethics board members) (*Alberta – 1; Ontario – 1*)	2

**TOTAL**		**55**

Study 3 consisted of 13 patients/health system users (one from every province and territory). Patients were identified and approached through various health networks, organizations and associations. In addition, 13 health policy makers representing different levels of government and different health care contexts (at least one policy maker from each province and territory) were recruited (table 4).

**Table 4:** Summary of Focus Group Participants: Policy Makers

*MACRO*	National level (*Canadian Medical Foundation, Canadian Nurses Association, Health Canada, Western Canada Waiting List Project*)	4
	
	Provincial Level (*Provincial Ministry of Health, Provincial Government (other than MOH)*)	3
*MESO*	Senior Management of Regional Health Authorities (*P.E.I, Manitoba, Alberta*)	5
	
	Senior Management Hospitals	1

TOTAL		13

Sample size was not formally calculated for any of the three studies since our goal was not to generate generalizable conclusions, but instead to describe characteristics of successful priority setting from the point of view of decision makers.

### Data Collection

Study 1 spanned three Delphi 'rounds'. Round 1 was conducted in May/June 2003 via email; the ethical framework 'accountability for reasonableness' (A4R) acted as the starting point for discussions [10]. (A4R was chosen as a starting point for discussions because it has traction among decision makers and it is an established framework of priority setting researchers internationally; moreover, it is a useful tool and a practical guide to develop, implement, and evaluate fair priority setting processes.)

Round 2 had all participants face-to-face; the input was a list of 39 items, generated from Round 1 (see appendix). Round 3 was conducted by email four months after Round 2. Results of Round 2 (now 14 succinct and prioritized items) were circulated; panelists were asked to make final suggestions and revisions to sharpen the list. Subsequently, the list was revised down to six items (table 5).

**Table 5:** Elements of Success – Results from 3 Studies

**Views of International Scholars and Decision Makers (Delphi)**	**Views of Canadian Decision Makers** **(1on1)**	**Views of Canadian Patients and Decision Makers (Focus Groups)**
(1) Improved Stakeholder Understanding (2) Acknowledgement of Appeals (3) Increased Stakeholder Acceptance and Satisfaction (4) Improved Decision Making & Social Learning (5) Shift in Resource Distribution (6) External Factors	(1) Explicit Process (2) Context Consideration (3) Consideration of Values (4) Inclusive Process (5) Effective Communication (6) External Guidance and/or Directives (7) Support a Learning Organization	(1) Integrated Process (2) Inclusive Process (3) Effective Communication (4) Education (5) Transparency of Process and Information (6) Consideration of Context (7) Consideration of Values (8) Recognized Shift/Change in Resources

Study 2 interviews were conducted in person or by telephone from July 2003 to May 2004 and used an interview guide based on previous research and relevant literature. All interviews were audio taped and transcribed – over 800 pages of transcripts were generated.

Study 3 was set around an existing event: an Alberta-based Provincial Health Ethics Network (PHEN) conference (April 2003). The study utilized this conference as a unique opportunity to bring together patients and policy makers in one location. All study participants participated in the PHEN conference. All focus groups were video taped and the discussions were transcribed. Observations were recorded by the researchers in field notes which provided context to the data analysis.

### Data analysis

Data from the three studies was first analyzed separately and then synthesized and analyzed in aggregate. Analysis was done using a modified thematic analysis that proceeded in two steps: open and axial coding [38]. In open coding, the data was read and then fractured by identifying chunks of data that related to a concept or idea. In axial coding, similar ideas were organized into overarching themes by grouping similar codes. The analysis was facilitated by, and culminated in, writing, which served as an important tool in formalizing elements and making explicit assumptions that influence data interpretation [39].

The validity of the findings was addressed in three ways [40]. First, two researchers (SS and DKM) coded the raw data to ensure accuracy and that one person's biases did not unduly skew the interpretation – differences were resolved through ongoing discussion. Second, all research activities were rigorously documented by the researcher to permit a critical appraisal of the methods [41]. Third, throughout all three studies, participants verified the reasonableness of the findings in "member checks" – participants were invited to read the results from the data analysis and comment on misinterpretations or missing information. Revisions from participants were incorporated into the findings, or where disagreement occurred, were discussed by the research team to determine further action.

### Research Ethics

All three studies received research ethics board approval from The Committee on the Use of Human Subjects of the University of Toronto. Where appropriate, written informed consent was obtained from each individual. All data was protected as confidential and available only to the research team. No individuals have been identified in reports without their explicit agreement.

## Results

### Synthesis of 3 Studies

When analyzed independently, the three studies provided insight into key elements which could define successful priority setting; together they provided 21 elements (table 5). In order to make one comprehensive list of elements of successful priority setting, we re-read and re-analyzed raw data to look for similarities; similar items were merged and an amalgamated list was created (e.g., within the views of Canadian decision makers, 'context considerations' and 'consideration for values' were merged; in the focus group list, 'consideration of context' and 'consideration of values' were merged; combined they created 'consideration of context and values'). Effort was made to ensure that the merged list captured the original description and meaning.

In the end, a list of ten elements was created (table 6). The research team created the element labels (left column) based on the results of the three studies; where possible, we used labels that were verbatim from the original raw data (i.e. participants themselves used the words).

**Table 6:** Merged List

	**Elements of the Conceptual Framework**	**Delphi**	**1-on-1**	**Focus Groups**
PROCESS	1. Stakeholder Engagement		x	x
	
	2. Explicit Process		x	x
	
	3. Information Management			x
	
	4. Consideration of Context & Values		x	x
	
	5. Revision or Appeals Mechanism	x		

OUTCOMES	6. Stakeholder understanding	x	x	x
	
	7. Shifted priorities/Reallocation of resources	x		x
	
	8. Improved Decision Making Quality	x	x	
	
	9. Stakeholder Acceptance &Satisfaction	x		
	
	10. Positive Externalities	x	x	

When there was disagreement or uncertainty about merging items (i.e. can they legitimately be combined, or should they remain separate), we went back to the original data and re-analyzed the individual and specific meaning of the element and how it originally emerged in the data. There were not many inconsistencies between the derived success elements in each of the three studies. Given the controversial nature of priority setting, this finding might seem out of place; however, it showed that there are common elements reasonable people can agree on [10]. By providing a forum to discuss priority setting, different stakeholders were able to come to some agreement on elements important to any priority setting activity. Further, the aim of this conceptual framework was to identify higher-level elements of success, about which there seems to have been a certain amount of consensus across healthcare settings and stakeholder groups.

There were some contradictions within study 2, between the focus groups (patients/health system users and decision/policy makers) mainly to do with procedural elements of priority setting. For example, patients were less concerned with procedural efficiency, but more focused on partnership in public consultation and education. Decision makers saw the importance of public consultation, but spent more time discussing the priority setting process, highlighting (among other things) the importance of efficiency.

We circulated the conceptual framework and an explanation of the elements using electronic communications to a selection of participants from the three studies as well as a group of interdisciplinary scholars, for their comments and refinements – a type of 'member check'. Across the studies, 15 participants were invited to comment. Additionally, eight scholars were asked to comment on the framework. Seven participants and all eight scholars replied via email with comments and questions of clarity. Most of the comments had to do with wording of the elements. For example 'information management' was clarified and further qualified as 'clear and transparent information management'. Another example: 'improved' was added to 'stakeholder understanding' to reflect the idea of change over time. Revisions were made accordingly.

Each element is important individually, but is also related to the others, thus forming a robust and comprehensive definition of successful priority setting in a broad conceptual framework; each of the ten elements is described below.

## Process Concepts

### 1. Stakeholder Engagement

Stakeholder engagement refers to an organization's efforts to identify the relevant internal and external stakeholders and to involve these stakeholders effectively in the decision-making process. This should include, at a minimum, administrators, clinicians, members of the public and patients. To ensure adequate engagement, identifying and engaging stakeholders should involve multiple techniques, such as round tables, open forums, departmental meetings. There should be a genuine commitment from the organization to engage stakeholders effectively through partnership and empowerment. Stakeholder engagement is also concerned with stakeholder satisfaction regarding the level of their involvement in the decision-making process.

### 2. Use of Explicit Process

An explicit process is one that is transparent not only to decision makers, but also to other stakeholders. Adhering to a predetermined process can enhance trust and confidence in the process. Transparency means knowing who is making the decision, how the decision will be made, and why decisions were made. Communication needs to be well coordinated, systematic and well-planned. All stakeholders (internal and external) should be probed for relevant information to the priority setting decisions and information should be communicated effectively using multiple vehicles (town-hall, departmental meetings, memos, emails, etc.).

### 3. Information Management

Information management refers first to the information made available to decision makers during the priority setting process, including what was used and what was perceived to be lacking. Second, information management considers how the information was managed, including how it was collected and collated. Relevant information includes, but is not restricted to: health outcomes data, economic data (such as cost effectiveness analyses), community needs assessment, current policies or policy reports, and the experiences of both clinicians and patients.

### 4. Consideration of Values and Context

Values and context are important considerations in any priority setting process, including the values of the organization, the values of staff within that organization, and the values of other stakeholders (such as patients, policy makers, politicians, and members of the community). The mission, vision and values of the organization should guide priority setting. Priority setting decisions should be based on reasons that are grounded in clear value choices, and those reasons should be made explicit. This also involves not only looking within the organization at previous priority setting decisions, but also observing what other healthcare organizations are doing. This would involve looking at organizations in the local community, at other healthcare organizations with similar mandates, as well as looking at the other levels of healthcare provision. Context is distinct from values and considers the organization's goals in the health care environment articulated in its strategic directions.

### 5. Revision or Appeal Mechanism

A revision process is a formal mechanism for reviewing decisions and for addressing disagreements constructively. It is important to have such a mechanism and to ensure its rules and requirements are communicated clearly ahead of time. The dual purposes of a revision process are to: 1) improve the quality of decisions by providing opportunities for new information to be brought forward, errors to be corrected, and failures in due process to be remedied; and 2) to operationalize the key ethical concept of responsiveness.

## Outcome Concepts

### 1. Improved Stakeholder Understanding

Stakeholder understanding implies more than basic knowledge of the process. It assumes stakeholders have gained insight into the priority setting (e.g. goals of the process, rationale for priority setting and rationale for priority setting decisions) and/or the organization (e.g. mission, vision, values, and strategic plan). As stakeholder understanding increases, stakeholder acceptance and confidence should also increase.

### 2. Shifted Priorities and/or Reallocated Resources

A successful priority setting process results in the allocation of budgets across portfolios, changes in utilization of physical resources (e.g. operating theatre schedules, bed allocations) or possibly changes in strategic directions. Effort that does not result in change may encourage the perception among stakeholders that the process was an inefficient use of time or mere window-dressing for pre-determined outcomes. A reaffirmation of previous resource allocation decisions (e.g. the previous year's budget) may, in some circumstances, be seen as a success.

### 3. Improved Decision Making Quality

Decision making quality relates to appropriate use of available evidence, consistency of reasoning, institutionalization of the priority setting process, alignment with the goals of the process and compliance with the prescribed process. It also captures the extent to which the institution is learning from its experience to facilitate ongoing improvement. This component is most obvious as subsequent iterations of priority setting are evaluated; where consistency and building on previous priority setting would be indicative of a successful process. Institutional learning, increased institutionalization of the priorities, more efficient decision making, more consistent decision making, or increased compliance with decisions (i.e. 'buy-in') are valuable, hard to achieve outcomes of successful priority setting. Institutional learning from its experiences facilitates ongoing institutional improvement, which appears as subsequent iterations of priority setting are evaluated.

### 4. Stakeholder Acceptance and Satisfaction

It is important to consider the satisfaction of all stakeholder groups, both internal to the hospital and external to the hospital (community groups/public and governmental health agencies/ministries of health). Successful priority setting leads to increases in satisfaction over multiple decision cycles. Stakeholder acceptance is indicated by continued willingness to participate in the process (i.e. 'buy-in') as well as the degree of contentment with the process. Stakeholders may be able to accept priority setting decisions, even if they may not always agree with the outcomes.

### 5. Positive Externalities

Positive externalities can act as a sort of check and balance, ensuring information is made transparent to stakeholders through various avenues, and/or establishing good practices for budgeting in other healthcare organizations. As an indicator of success, externalities may include positive media coverage (which can contribute to public dialogue, social learning, and improved decision making in subsequent iterations of priority setting), peer-emulation or health sector recognition (e.g. by other health care organizations, accreditation bodies, etc), changes in policies, and potentially changes to legislations or practice.

## Discussion

The primary purpose of this study was to develop a conceptual framework for successful priority setting. This research has helped elucidate elements of successful priority setting which can be used to assist organizations in their priority setting efforts.

Priority setting is complex, difficult, contentious and often controversial. Developing a conceptual framework is a necessary first step to approaching the evaluation of successful priority setting. The findings of the three studies described here have been synthesized into a conceptual framework which aims to provide guidance to decision makers (and other stakeholders) in better understanding successful priority setting.

The conceptual framework contains ten elements germane to successful priority setting. It is an advance in knowledge because it is the first attempt to comprehensively describe elements of successful priority setting from the point of view of stakeholders. It provides a way of thinking about successful priority setting and the considerations, or components, essential to achieving successful priority setting. It also provides a basis for decision makers to think specifically about successful priority setting and how to achieve it. Finally, it offers a common language for decision makers and stakeholders, within and between institutions, to discuss successful priority setting.

This research is complementary to previous studies that identified pieces of successful priority setting, and it builds and expands upon these previous works by describing a broad range of stakeholders' views about successful priority setting and synthesizes them into one conceptual framework that can be used by decision makers to improve priority setting. Further, this framework focuses both on process and outcomes of priority setting – other descriptive frameworks (e.g. accountability for reasonableness) focus only on the fairness of the process.

The ten elements identified in this framework are interconnected and often interdependent, it is difficult to use these elements in isolation. Elements were not weighted since there was no empirical evidence to suggest one element was more important than another. All elements are relative – that is, as conditions, they may be more or less met, and each may be improved.

Although the ten elements are not directly derived from moral theory, they hold normative relevance because they are derived from overlapping consensus of empirical observations involving the participants' reported values. Many of the participants were actual priority setting decision makers who are motivated to improve priority setting because they are directly involved in it. It is important to distinguish here between normative versus positive. This 'fact/value distinction' differentiates statements about what is the case from statements about what ought to be the case. Facts are descriptive, telling us what was done; values are prescriptive, telling us what should be done. The value-relevance of this study comes from the participants' values – i.e. their normative reasoning – not from the data analysis. In this research, we have 'described' participants' views; the participants have provided what they thought 'should be'.

The ten elements of successful priority setting in our framework have been organized into two types: process concepts and outcome concepts. Traditionally in health care, outcome measures refer to health outcomes (e.g. morbidity and mortality) in a selected population. However, the respondents in our three studies did not mention health outcomes as an element of successful priority setting. Thus, our framework does not include health outcomes in its list of priority setting outcomes. Our framework may then be criticized for not including health outcomes. A critic may ask: In an organization dedicated to health care, can a priority setting exercise that results in poorer health outcomes really be considered successful? Future scholarship should examine these divergent views. Health outcomes may be influenced by priority setting decisions, but are also influenced by a myriad other factors, such as quality of care. Outcome measures, such as mortality rates, may be helpful in evaluating the success of a healthcare organization, but there are many complicating factors – e.g. What about organizations that deliberately treat very complex cases? According to our respondents, achieving priority setting success is possible by focusing directly on priority setting outcomes, such as improved stakeholder understanding, shifted priorities, improved decision making, stakeholder acceptance, and positive externalities. Ultimately, we suspect that future research may find strong associations between health outcomes and priority setting outcomes such as stakeholder acceptance.

Our study is supported by previous studies that have reported on pieces of our framework [2,5,6,20]. While other frameworks exist to help decision makers with priority setting, our framework is more comprehensive. For example, Gibson, Mitton et al showed that while PBMA can be effective, it is not comprehensive; they suggested combining PBMA with A4R to achieve optimal benefits with available resources [42]. This framework is an advance because it was derived directly from conversations with people involved in priority setting about priority setting, and because it is the first time they have all been connected together in a comprehensive framework (table 6).

Our study builds and expands upon previous works by, for the first time, describing a broad range of stakeholder's views about successful priority setting and synthesizing them into one conceptual framework that can be used by actual decision makers to improve priority setting.

## Limitations

The primary limitation of this research is its generalizability. The results of this research reflect the views of a wide range of key stakeholders, but most are from the Canadian health system, and they may not represent the views of stakeholders in other countries or cultures. It is also important to note that each country may have additional contextual elements of success. Moreover, participants were not sampled by a statistical method designed to yield generalizable results. The sampling technique was designed to probe a range of perspectives. Further research is required to determine the wider applicability of the concepts described here. Second, it is possible that the views provided by participants were shaped by social desirability bias, and responses given in the interviews might not correspond to what their organization actually does. However, we found no glaring inconsistencies between the interview data and the documentary support.

## Conclusion

Using an innovative and robust mixed-methods approach, we have created a framework which attempts to provide much needed guidance to decision makers (and other stakeholders) to begin to improve the success of priority setting. Health care decision makers need guidance to set priorities. This study has helped elucidate the elements of successful priority setting which can be used to assist organizations in their priority setting efforts. Further research is needed to determine how best to utilize them to evaluate success of priority setting.

## Competing interests

The authors declare that they have no competing interests.

## Authors' contributions

SS was the primary analyst and principal author of the manuscript. DKM conceived the research, was involved in data collection and analysis, and was co-author of the manuscript. RU and PAS were involved in study conception, analysis and drafting the manuscript. All authors read and approved the final manuscript.

## Appendix DELPHI ROUND ONE LIST OF ITEMS

### Directly Related to A4R

#### Relevance

1) Assessments of the health needs or other interests of the affected populations have been determined and documented. Other interests could take into account concessions on health needs for other gains or advantages (job security, education) as result from collective bargaining or political processes.

2) Representatives of different stakeholders groups are represented and meaningfully participate in the allocation decision-making process.

3) Data or generally accepted opinion exist that support specific allocation policies and management practices.

4) No policies or management practices (e.g., requirements for patients or providers) are in place that can frustrate access to the allocated health care services either purposely or inadvertently.

5) A systematic search and evaluation of evidence

• Conformance with evidence would require expert judgment

• The quality of decisions should be higher because rationales are required, there is less scope for decisions to be based on considerations other than the available evidence e.g. lobbying and political pressure, though lobbying will still occur.

6) Wide professional consultations

#### Publicity

7) Communication materials and mechanisms made available by policy makers, and by surveys of stakeholders and direct observation approaches.

8) Decisions are public and accessible

9) Reasons are given in non-technical language

#### Appeals

10) Policies, rationales, and requirements can be revised as made necessary by changes in objectives to providing allocations or new information or arguments that have a bearing on allocation decisions.

11) Policies and procedures in place addressing surveillance needs to determine when changes are necessary to general allocation policies and to adjudicate individual requests from stakeholders for revisions in general policies or individual decisions.

12) Documentation exists showing responses to new information or stakeholder requests for changes in policies or practices

#### Enforcement

13) Mechanisms exist that ensure the processes are available and function properly

14) Governmental regulatory requirements for compliance to processes.

15) Internal policies and procedures (including auditing functions) to ensure compliance.

16) Voluntary arrangements with independent third-parties exist to assess compliance with processes and/or to adjudicate stakeholder requests for changes in policies or for appeals of individual decisions.

#### Other forms of outcome indicators

Available through interested observers such as governmental agencies, courts, news media, and cultural apparatus; could include, but not be limited to the following:

17) Qualitative and quantitative measures of federal and local legislation and regulation targeting problems meant to be addressed by the main ideas of accountability for reasonableness

18) Qualitative and quantitative measures of complaints and grievances about health care service allocation policies and management practices brought by stakeholders in the process

19) Number of appeals submitted for unavailable health care services that can be tied to insufficient conformance to the main ideas of accountability for reasonableness

20) The number of lawsuits filed and the size of awards provided for problems that correspond to the main ideas of accountability for reasonableness

21) The number and nature of news media accounts of problems with health care service allocation policies and management practices

22) The frequency and nature of content in common cultural media (plays, movies, books)

23) Principles or criteria are explainable and justifiable to lay audiences need to have at their core the overriding responsibility to make decisions consistent with the public's health needs as well as available resources – both present and future.

24) Evaluation that has structure and is somewhat generic

• An evaluation framework for measuring effectiveness of the given priority setting process that provides structure for evaluation but is also generic enough to be adapted in the local context

• Tool provides guidance but is at the same time not overly prescriptive

25) Resource inequalities are compensated

• Re-allocation of resources; improved patient outcomes

26) Relevant Stakeholders: consideration of the differing roles of governing bodies, executive management, operational management, and (in some situations) physicians and other health care professionals – but also alignment with the decision-making structure of the affected organization (who gets to decide what?).

27) The organization must be inclusive enough for the participation of key stakeholders, to be accepted by all parties; The organization must be exclusive enough to reach a limit-setting decision within reasonable time and resources; All key stakeholders have equal access and voice.

28) Stakeholder understanding: greater knowledge of why decisions have been made

29) Impact on stakeholder understanding of limits and their rationales

• Measured in surveys in natural experiments

• Measured in use of web pages or other devices for explaining limits, eg: of pharmacy benefits

30) Satisfaction of the participants: self-rated usefulness by participants; important to draw on the judgments of decision makers themselves and of key stakeholders; whether decisions 'felt fair' – as assessed by decision makers and stakeholders, and in the context of what has been achieved in other settings.

31) Policies and mechanisms in place to make affected populations aware of

• Objectives to providing covered health care services

• Health services available and specific conditions/requirements

• Mechanisms available that facilitate access to covered health services, including appeals processes

• Rationales for allocations, conditions, and requirements

32) Degree to which main ideas become embedded in culture: improvement could be measured by the nature and number of enhancements to the original process

33) Enhancement of market perception: of provider in situations where some providers promote themselves as abiding by A4R

34) High degree of stakeholder acceptance

35) High degree of reasonable public acceptance

### Indirectly Related to A4R (but relevant to effectiveness)

36) There needs to be clear objectives/purpose: decision makers need to have clear objectives upon which they agree.

37) Commitment to implementation: without a commitment to implementation/follow-through based on the results, the process is incomplete and its credibility may be undermined for any subsequent use.

38) Maximization of benefits and minimization of opportunity costs

39) Effectiveness measured by efficiency:

• An efficiently timed process that provides for meaningful involvement without demanding excessive time or effort.

• a lengthy time for stakeholder involvement, etc., crucial energy and sustained knowledge/understanding and commitment can be compromised.

## Pre-publication history

The pre-publication history for this paper can be accessed here:


